# Patient handover – the poor relation of medical training?

**DOI:** 10.3205/zma001227

**Published:** 2019-03-15

**Authors:** Barbara Hinding, Nicole Deis, Maryna Gornostayeva, Christian Götz, Jana Jünger

**Affiliations:** 1 The German National Institute for state examinations in Medicine, Pharmacy and Psychotherapy, Mainz, Germany

**Keywords:** medical education, interprofessional education, handover communication, teaching

## Abstract

**Objective: **The handover of patients to medical colleagues and to members of other professional groups is a central task in the medical care process for patient safety. Nevertheless, little is known about teaching and testing on the subject of handing over. The present article therefore examines the extent to which handover is the subject of teaching and examinations at medical faculties in Germany.

**Methodology: **In 31 medical faculties the teachers were asked about the implementation of the NKLM learning objectives in the area of communication. The survey was conducted within the framework of group interviews with lecturers, in which it was determined whether each learning objective of the NKLM (National Competency-based Catalogue of Learning Objectives in Medicine) on the subject of communication, is explicitly taught in lectures and examinations at the respective faculty.

**Results: **The learning objective "transfer to medical colleagues" is covered by 19 faculties, while the learning objective of interprofessional transfer is covered by 14 faculties. There are examinations for transfer to medical colleagues and interprofessional transfer at two faculties. There is a highly significant relationship between the total number of communicative learning objectives that are put into practice in a faculty and the coverage of the learning objectives for handover.

**Conclusions: **In the field of communications, the subject of handover is less frequently taught at the faculties and, more importantly, it is less frequently examined than other NKLM contents. This is particularly evident in the interprofessional area. The subject is more likely to be taught as a handover between physicians, while the interprofessional interfaces attract less attention. In terms of patient safety, it would be desirable to give a higher priority to the subject of handover. An inter-faculty exchange and the inclusion of the subject of intra- and interprofessional transfer in state examinations could give the implementation process at the faculties a decisive impetus.

## Zusammenfassung

**Zielsetzung:** Die Übergabe von Patient*innen an ärztliche Kolleg*innen und an Angehörige anderer Berufsgruppen ist eine für die Patientensicherheit zentrale Aufgabe im Versorgungsprozess. Dennoch ist über Lehre und Prüfung zum Thema Übergabe nur wenig bekannt. Im vorliegenden Beitrag wird daher untersucht, inwieweit Übergabe Gegenstand von Lehre und Prüfungen an den medizinischen Fakultäten in Deutschland ist.

**Methodik:** An 31 medizinischen Fakultäten wurden die Lehrverantwortlichen zur Umsetzung der NKLM-Lernziele im Bereich Kommunikation befragt. Die Erhebung erfolgte im Rahmen von Gruppeninterviews mit Lehrverantwortlichen und Dozent*innen, in denen für jedes Lernziel des NKLM zum Thema Kommunikation ermittelt wurde, ob es an der jeweiligen Fakultät in Lehrveranstaltungen und Prüfungen explizit umgesetzt wird.

**Ergebnisse**: Das Lernziel „Übergabe an ärztliche Kolleg*innen“ wird an 19 Fakultäten abgedeckt, das Lernziel zur interprofessionellen Übergabe an 14 Fakultäten. Prüfungen zur Übergabe an ärztliche Kolleg*innen und zur interprofessionellen Übergabe gibt es jeweils an zwei Fakultäten. Dabei besteht ein hochsignifikanter Zusammenhang zwischen der Gesamtanzahl umgesetzter kommunikativer Lernziele an einer Fakultät und der Abdeckung der Lernziele zur Übergabe.

**Schlussfolgerungen: **Das Thema Übergabe wird an den Fakultäten seltener gelehrt und vor allem seltener geprüft als andere NKLM-Inhalte im Bereich Kommunikation. Ganz besonders zeigt sich das für den interprofessionellen Bereich. Das Thema wird eher als Übergabe zwischen Ärzt*innen unterrichtet, während die interprofessionellen Schnittstellen weniger Beachtung finden. Im Hinblick auf Patientensicherheit wäre es wünschenswert, dem Thema Übergabe eine höhere Priorität einzuräumen. Ein interfakultärer Austausch und die Aufnahme des Themas intra- und interprofessioneller Übergabe in die Staatsexamina könnten den Implementierungsprozess an den Fakultäten entscheidend voran bringen.

## 1. Background and problem definition

Patient safety is an important goal for all professional groups involved in patient care. Nevertheless, adverse events take place in everyday clinical practice. Many of them could be avoided if collaboration and communication were given more attention. Communication problems and lack of communication in the team can lead to many undesirable events that endanger the patient [1]. Difficulties in communication are mentioned as a cause in nearly two-thirds of all errors [2].

Reasons for suboptimal communication may be seen in the presence of situation-specific conflicting goals. Staff shortage and time pressure, high work intensity and heavy workload as well as individual motivations restrict the subjective perception of the possibilities of action. Work requirements and resources must be reconciled in order to do the work as efficiently as possible. This often happens at the expense of communication and security. Hollnagel [3] describes this conflict as the "ETTO principle". In essence, it describes the trade-off between efficiency and care. Therefore, organizations and the people working in them have to balance a resource-economical approach with careful task execution, since both are not optimally possible at the same time. When performance requirements are high, caution and accuracy are reduced. If safety is to be given top priority then efficiency expectations must be reduced.

The concept of “accepted level of risk” [4] provides a way out of this dilemma. It is an intrinsic threshold that adjusts hazards in a workstation. This is largely dictated by the organization and its surrounding system (e.g. safety regulations, warnings, sanctions) and controls individual perception and behaviour. This means that employees form their accepted level of risk in relation to the system and internal setpoints. One possible solution, therefore, is to change this culture in such a way that risk-taking is reduced. An important step in this direction is to communicate the importance of patient safety and related communication skills required in this area, during education itself.

In Germany, numerous measures have been introduced in recent years to promote patient safety, to implement the recommendations of the European Union on patient safety in Germany [http://www.cirsmedical.de cited 2018 Jan 15], [http://www.aps-ev.de cited 2018 Jan 15] [5] e.g. the CIRSmedical Germany, the Patient Safety Action Alliance and the initiative of the German Medical Association. Special attention is given to the issue of patient handover. Handover of patients, as defined by the British Medical Association (2004), refers to “the transfer of professional responsibility and accountability for some or all aspects of care for a patient, or group of patients, to another person or professional group on a temporary or permanent basis” [6].

Above all, it serves to forward clinically relevant information in order to ensure continuous care for patients. Studies show that structuring the handover process improves its quality and consistency and has a positive impact on communication, teamwork and patient safety [7], [8], [9]. 24% of nurses and 39% of physicians rate handoversas inefficient and not well structured. 55% of nurses and 32% of the medical profession complain about problems in communication and coordination between the medical service and nurses [10].

Introducing structured submission schemas reduces the frequency of treatment errors, improves the quality of treatment and patient safety, and increases patient and employee satisfaction [11], [12], [13], [14], [15].

The transfer of patients to colleagues, to nursing staff and to members of other professional groups, e.g. Physiotherapy, is an essential part of the medical profession. Younger doctors are confronted with this task during their first days in a clinic. Accordingly, this competence should be attained during the training itself. In its decision of 2014 the Conference of Health Ministers also calls on the legislator to “give greater weight to patient safety as a subject of study and examination” in the occupational laws for the health professions [16]. Accordingly, the Society for Medical Education's Patient Safety Committee has issued its own learning objectives catalogue with 68 learning objectives on patient safety [17]. This addresses transfer in two learning objectives, which can also be found in the chapter “Medical Interviewing” of the National Competency-based Catalogue of Learning Objectives in Medicine (NKLM) [18], [19].

Based on the learning objectives of the NKLM chapter “Medical Interviewing”, a National Longitudinal Model Curriculum on Communication was developed in the project “Longkomm” (Longkomm: Communicative Competences of Physicians in Oncology – Development of a longitudinal, oncological core curriculum based on the implementation recommendations of the National Cancer Plan), funded by the Federal Ministry of Health (BMG) [19], [20], [21]. With measure <8> the objective of the master plan for medical studies 2020 is to incorporate the “National longitudinal communication curriculum in medicine” in the curricula of universities and to develop special examination formats for it [22].

The present study investigated how many faculties teach and examine the subject of intra- and interprofessional handover:

- How many faculties are implementing both or at least one of the two learning objectives?
- How well are learning objectives covered in comparison to all other communication-related learning goals?
- Is there a correlation between the learning objective coverage on the subject of handover and the learning objective coverage for the other communications learning objectives?

## 2. Procedures and methods

From July 2013 to May 2015, the Longkomm project carried out a survey of teaching and examinations in the area of communicative competences in medical studies at medical faculties in Germany in order to determine the extent to which the learning objectives defined in the NKLM have been implemented and where there is a need for action.

36 medical faculties were invited to participate.

First of all, all faculties were contacted to obtain local coordinating contact persons for communications teaching. Following this, the teachers responsible for communication at the location were identified together with these coordinators and an overview of the courses and examinations with communication-related content was created with them using structured, telephonic interviews. Courses and examinations were only included if aspects of the medical discussion were an explicit part of the lesson or subject of the examination.

Four faculties did not take part in the survey because either no central faculty contact could be determined, or an actual analysis was not possible due to a comprehensive revision of the communication curriculum during this period, or participation was not (yet) considered useful due to intra-faculty discussions regarding the NKLM. Of the 32 faculties covered, 1 faculty had to be excluded from the following analyses due to missing data.

The NKLM contains two learning objectives on the subject of “handover”:

- LO 6.1.1: Carrying out an oral or telephone handover of patients to medical colleagues
- LO 6.1.2: Carrying out an oral or telephone handover of patients to members of the nursing or other healthcare professions using appropriate terminology.

In workshops at the faculties, these two learning objectives, along with all other communication-related learning objectives in the NKLM, were mapped. To the workshops, the teaching staff of all teaching sessions and exams on communication that were recorded previously during the telephone interviews were invited. For each of the courses identified and each examination with communicative content, it has been determined whether it is optional or mandatory and which communication-related learning objectives it addresses. The basis for this was a structured instrument, in which for each of the learning objectives in NKLM chapter 7 “The doctor as a communicator” and 14c “Medical discussion” it was to be recorded whether it was covered or not. A learning objective was considered covered for a course or exam if it was mostly (>60%) dealt with in the course or evaluated in an examination.

The descriptive statistical evaluation was done using SPSS version 24. For each of the two learning objectives it was first determined how many faculties covered the objective in courses and examinations. Furthermore, the mean, standard deviation and the range (minimum and maximum value) were determined for all participating faculties.

In addition, the question was whether faculties that already have an extensive range of courses in the field of communication, teach more methods of safe handover than faculties that have just begun to implement their curriculum. In order to clarify the question of this relationship between coverage of learning objectives of the subject of transfer and coverage of learning objectives of communication, the Pearson correlation coefficient was determined. In assessing the effect size, a convention described by Cohen (1988) [23] was followed, according to which a correlation coefficient of r=0.1 is a low correlation, r=0.3 is a moderate correlation and r=0.5 is a high correlation.

## 3. Results

### 3.1. Courses and examinations with communicative content

The following section gives an overview of the number of courses and examinations with communicative content. Data from 623 courses and 162 examinations with communicative content was collected from the 31 participating faculties. Data pertaining to the implementation of the NKLM learning objectives was collected from 506 of these courses (approx. 81%) and 99 of these examinations (approx. 61%). It showed that the German medical faculties have an average of 20.1 courses and 5.2 examinations with communicative content (see table 1 (Tab. 1)). Of these, 15.1 courses are mandatory and 4.7 are optional while in case of examinations, 4.4 are mandatory and 0.8 are optional.

In addition, a great deal of heterogeneity between the faculties became apparent. The range of information available is from 5 to 91 courses and 0 to 15 exams (see table 1 (Tab. 1)).

#### 3.2. Courses and examinations on the subject of “handover”

Data on learning objectives LO 6.1.1: Oral or telephone handover of patients to medical colleagues and LO 6.1.2: Oral or telephone handover of patients to members of the nursing or other healthcare professions using appropriate specialist language, is available to us from all the 31 faculties.

Learning objective 6.1.1 on transferring patients to colleagues was covered in mandatory courses at 19 faculties (61%). For learning objective 6.1.2 – interprofessional handover – there were mandatory courses at 14 faculties (45%). Out of the 31 faculties surveyed, 19 offered courses for LO 6.1.1 or LO 6.1.2.

On average, this results in a value of 1.6 courses per faculty for learning objective 6.1.1 handover between colleagues, and for learning objective 6.1.2 interprofessional handover a value of 0.7 courses per faculty. For the learning objective on handover between colleagues, the number of sessions ranges from one to eight at a faculty. For interprofessional handover there are a maximum of four sessions at one faculty (see table 2 (Tab. 2)).

Examinations on the subject of handover are significantly less frequent than teaching sessions. The subject of intra- and interprofessional handover is examined at only two faculties. More than 90 percent of the faculties do not conduct examinations on the learning objectives.

On average, this results in 0.1 examinations per faculty on the subject of handover for both learning objectives.

#### 3.3. Correlation between the number of learning objectives implemented overall and the learning objectives for handover 

There is a high and statistically significant correlation between the learning objective “handover to a colleague” and the number of learning objectives achieved in the area of communication. In the case of the examinations too, there is a correlation, but it is lower in value and has a lower level of significance (see table 3 (Tab. 3)).

## 4. Discussion

The results show that the courses on the subject of handover offered at the surveyed faculties are less frequent than other courses in the field of medical consultation. Thus, patient handover as a learning objective has not the priority that would be desirable in terms of patient safety. This is in conformance with another finding from the Longkomm project [22], according to which there are pronounced differences in the coverage of areas of competence from the NKLM.

In competence area 6 (other media channels and settings), which also includes transfer, the learning objectives were found to have the lowest level of coverage. It seems that in many places the doctor-patient conversation is the primary focus of training in communication, whereas the intra- and interprofessional interfaces have not been very well portrayed as yet.

It is also interesting to note the difference between the learning objective of transferring to colleagues, which is taught at about 60% of the faculties, and the learning objective of interprofessional handover, which is taught at only about 45% of the faculties. Patient handover is therefore taught more as a transfer between doctors while the interprofessional setting receives less attention.

The correlation found between the total number of courses and examinations on communication and the courses and examinations on the learning objectives for handover may be interpreted such that the faculties have progressed at different stages in the development and implementation of their communication curricula. Especially at the beginning of curricular development, the focus seems to be more on general competencies (e.g. from the area of competence 1 concepts, models and general principles) than on more specific settings such as patient handover.

Furthermore, it is not necessarily the case that teaching and assessing handover go hand in hand. The finding that only two faculties out of 31 assess handover speaks for itself. Even in the written state examination, the subject of handover does not exist as an examination subject. This result is worrying given that examinations are important drivers of students' learning activities. The issue of handover being a weak point of patient safety requires more attention and greater importance at this point. However, the reported findings are also subject to uncertainties. Not all the relevant courses could possibly be determined, as the selection of interviewees followed predominantly social heuristics. At the faculties, contact persons were sought, who then identified the teachers and instructors who teach communication. The scope of this group varied according to the faculty. Often it was a large number, so perhaps not all could be contacted. Furthermore, not all the teachers involved, participated in the workshop. Although telephone surveys were made here, it can be assumed that not all of them could be contacted. Perhaps, it may paint a more negative image than reality.

The statement that a course contains at least 60% communicative content is subjective and subject to bias. Assuming that the workshop participants respond in a socially desirable manner, the proportion of communication could have been overestimated from time to time.

However, the large number of recorded courses and examinations suggests that the figures represent, at least in part, the deficit in the mediation of inter- and intra-professional handover.

With regard to patient safety, it becomes apparent that there is a great need for action in medical education on the basis of the described findings. Offers of support are needed for promoting the implementation process at the faculties. Kiesewetter et al. give tips and ideas for the sustainable anchoring of a longitudinal curriculum on patient safety [24]. One way to promote networking and cooperation is to actively disseminate examples of good practice for teaching and examinations. [https://www.medtalk-education.de/toolbox/ for instance, provides an exchange platform for this with the tool box [25]. Lecturers provide their solutions in a structured form in a protected area, so that they can be adopted by others.

However, it must be borne in mind that teaching and examining patient handovers are no guarantee that the students will actually perform more effective and structured handovers later on in their professional practice. In professional practice, handover is not only dependent on what is explicitly taught and examined during the education but is to a great extent influenced by implicit factors (“hidden curriculum”) such as learning on the model. From the Anglo-American English-speaking world, however, it is known that the effects of training in patient handover are not only short-term, but that one year after the training the students carry what they have learned from the simulation to the clinical setting [26].

Another opportunity is the reorientation of the state examinations required in the Master Plan for Medical Studies 2020. Jünger [27] describes – in her proposal for the redesign of the third section of the medical examination – a bed-side clinical examination with an inpatient and an outpatient, in which both a handover to colleagues and an interprofessional handover are included. In order to optimally prepare students for the examination and subsequent professional career, it is necessary to integrate the teaching and examination of structured intra- and interprofessional handover into the curricula. In terms of patient safety, this could mean a huge step forward.

## Acknowledgements

Our thanks go to all who contributed to the success of the Longkomm project. In particular, we thank all those involved in the faculties who, as contact persons and coordinators or instructors and lecturers, have made the information necessary for the curricular mapping of communication accessible and have given us an insight into their teaching. We would also like to thank all employees working on the “Longkomm Project” who have mastered the elaborate data collection and evaluation in the allotted timeframe with great commitment, perseverance and care.

## Funding

Special thanks are also due to the Federal Ministry of Health (BMG) for funding the project within the framework of the National Cancer Plan (grant number ZMVI5 2514FSB216), without which it would not have been possible.

## Competing interests

The authors declare that they have no competing interests. 

## Figures and Tables

**Table 1:**
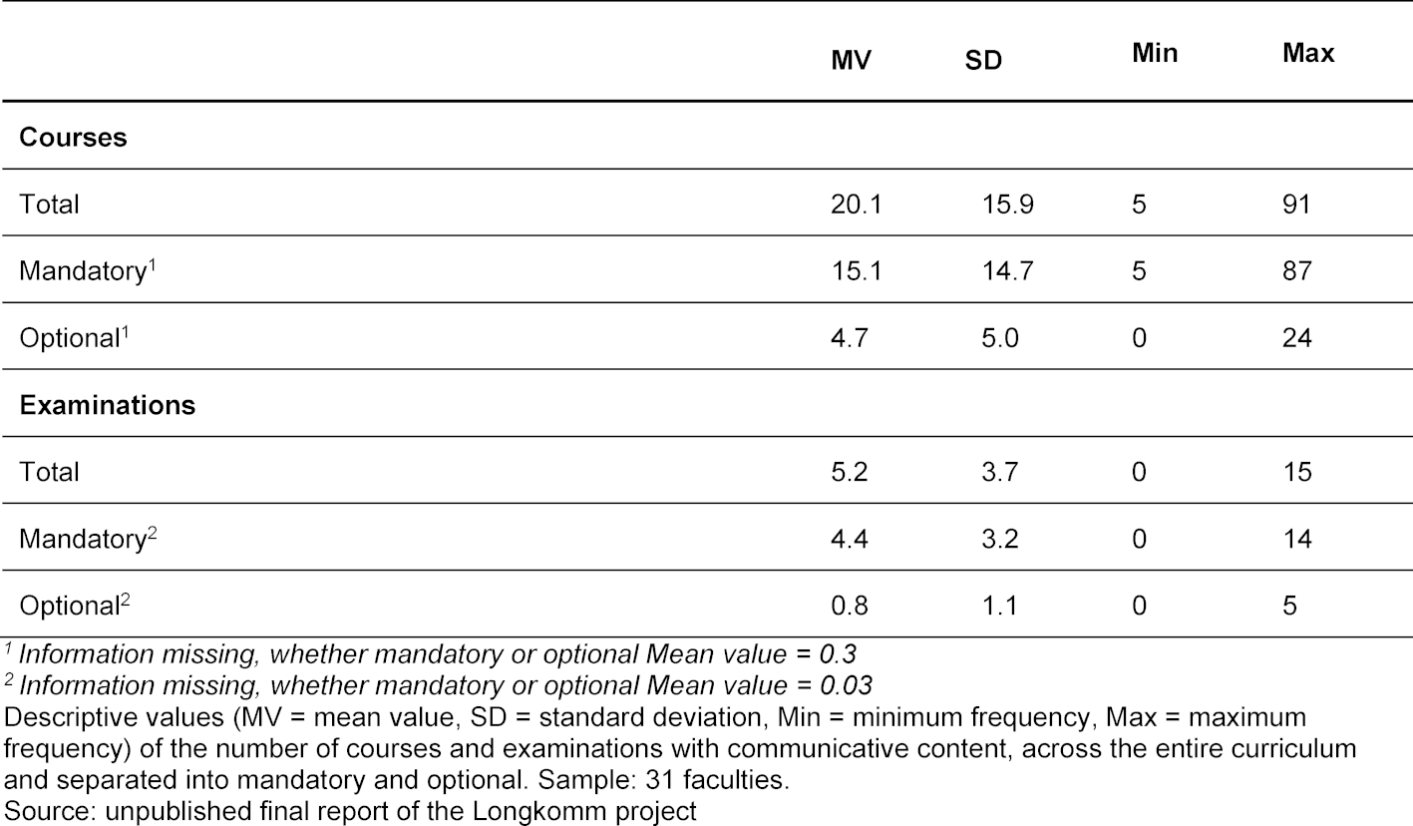
Average number of courses and examinations for each faculty with communication-related content

**Table 2:**
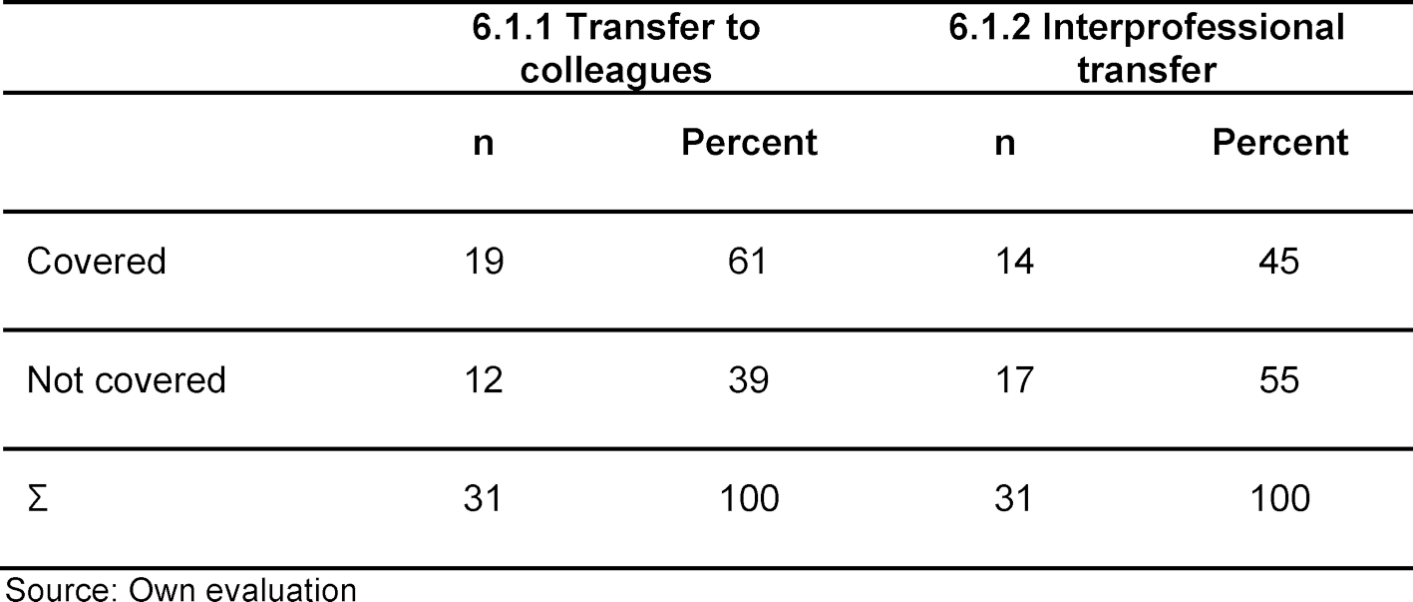
Proportion of faculties with courses on intra- and interprofessional transfer

**Table 3:**
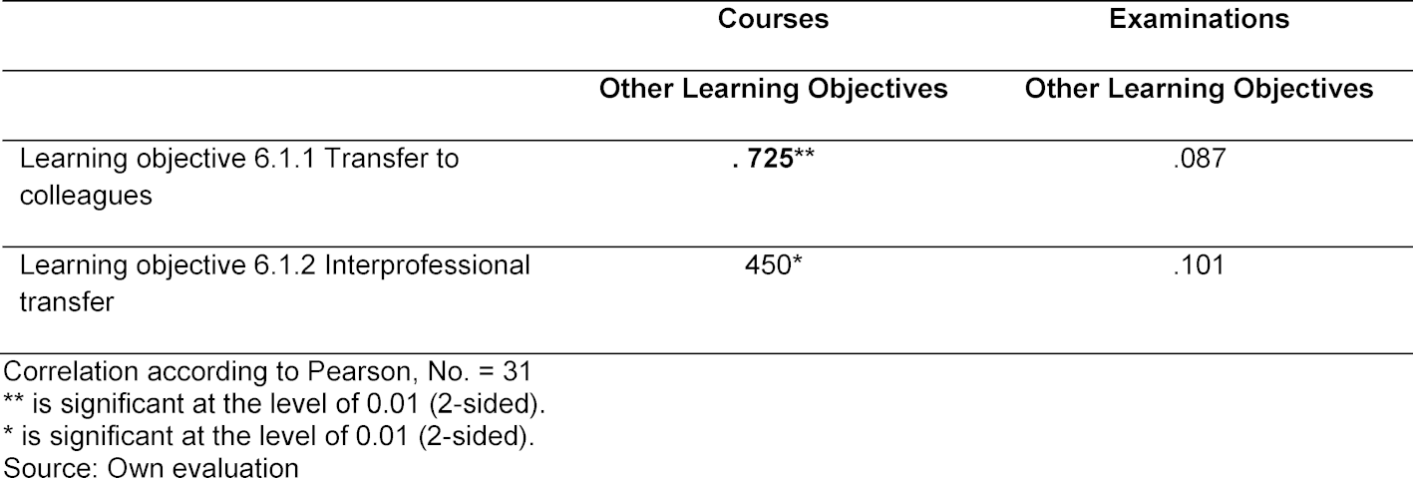
Relationship between learning objectives for transfer and other learning objectives for communication
